# Stochastic Simulations on the Reliability of Action Potential Propagation in Thin Axons

**DOI:** 10.1371/journal.pcbi.0030079

**Published:** 2007-05-04

**Authors:** A. Aldo Faisal, Simon B Laughlin

**Affiliations:** Department of Zoology, Cambridge University, Cambridge, United Kingdom; University College London, United Kingdom

## Abstract

It is generally assumed that axons use action potentials (APs) to transmit information fast and reliably to synapses. Yet, the reliability of transmission along fibers below 0.5 μm diameter, such as cortical and cerebellar axons, is unknown. Using detailed models of rodent cortical and squid axons and stochastic simulations, we show how conduction along such thin axons is affected by the probabilistic nature of voltage-gated ion channels (channel noise). We identify four distinct effects that corrupt propagating spike trains in thin axons: spikes were added, deleted, jittered, or split into groups depending upon the temporal pattern of spikes. Additional APs may appear spontaneously; however, APs in general seldom fail (<1%). Spike timing is jittered on the order of milliseconds over distances of millimeters, as conduction velocity fluctuates in two ways. First, variability in the number of Na channels opening in the early rising phase of the AP cause propagation speed to fluctuate gradually. Second, a novel mode of AP propagation (stochastic microsaltatory conduction), where the AP leaps ahead toward spontaneously formed clusters of open Na channels, produces random discrete jumps in spike time reliability. The combined effect of these two mechanisms depends on the pattern of spikes. Our results show that axonal variability is a general problem and should be taken into account when considering both neural coding and the reliability of synaptic transmission in densely connected cortical networks, where small synapses are typically innervated by thin axons. In contrast we find that thicker axons above 0.5 μm diameter are reliable.

## Author Summary

Neurons in cerebral cortex achieve wiring densities of 4 km per mm^3^ by using unmyelinated axons of 0.3 μm average diameter as wires. Many axons (e.g., pain fibers) are thinner. Although, as in computer chips, wire miniaturization economizes on space and energy, it increases the noise introduced by thermodynamic fluctuations in a neuron's “protein transistors,” voltage-gated ion channels. We investigated how well the relatively small number of ion channels found in the membranes of tiny axons propagate the brain's universal signal—the action potential. We built a stochastic model that incorporates the random behavior of individual ion channels and found noise effects much larger than previously assumed, because standard stochastic approximation techniques (Langevin) break down because single channels can produce whole-cell responses. Channel noise destroys information encoded in the timing of action potentials, by randomly varying the speed of conduction, and produces a novel mode of transmission, stochastic microsaltatory conduction. Ion channel populations retain memory of previous activity in the distribution of channel states, causing action potential reliability to vary with context. The effects and general relationships identified here will govern other cell-signaling systems that rely on inherently noisy protein switches to propagate signals, either for intracellular communication (Ca^++^/cAMP waves) or in nanotechnology.

## Introduction

Nervous systems use the action potential (AP) to send information rapidly and reliably along axons. The reliability of the AP is an essential prerequisite for encoding, transmitting and computing neural information [1], e.g., the precision of AP arrival times (“spikes”) is behaviorally relevant on the order of 1–10 ms in many species [2], and cortical neurons have specialized to detect coincident arrival of APs on the order of milliseconds [3].

The AP is mediated by voltage-gated ion channels whose gating behavior is subject to thermodynamic fluctuations which introduce a source of electrical noise in neurons, channel noise [4,5]. This channel noise is an inescapable part of the AP signaling mechanism. Previous modeling work showed that channel noise explained threshold fluctuations at individual Nodes of Ranvier and the reliability of spike initiation in membrane patches [6–9]. In vitro experiments related membrane potential fluctuations in dendrites and soma to channel noise [10–13], suggesting that fluctuations could indeed affect spike initiation reliability [14]. In an elegant application of the dynamic-clamp technique, channel noise was shown to be essential for generating the oscillatory behavior of entorhinal cortex slices [15,16].

In general, ion channels affect membrane potential in proportion to the membrane input resistance. In axons, this leverage increases as a power-law as axon diameter decreases (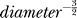 [17–19]). The thinner the axon, the stronger channel noise effects will be. Because the cable properties of thin axons shield them from dendritic and somatic sources of variability, such as synaptic input, resistive noise sources are orders of magnitude smaller than channel noise [20,21]. Other sources of variability (ephaptic coupling, input through axo-axonic synapses, and gap-junctions) are, unlike channel noise, not inherent to AP signaling and not, therefore, common to all axons. Thus, channel noise is likely to be the dominant source of electrical noise in unmyelinated axons below 0.5 μm diameter [19].

We have previously shown that channel noise causes AP communication to break down in axons and soma below 0.1 μm and 3 μm diameter respectively—a general limit to neuron size matched by anatomical data across species [19]. Axons in many important pathways operate close to or just above this limiting diameter, such as cerebellar parallel fibers (average diameter 0.2 μm [22]), C-fibers implicated in sensory and pain transmission (range 0.1–0.2 μm [23]), and pyramidal cell axon collaterals (average diameter 0.3 μm [24]) forming the local cortico–cortical connectivity with wiring densities up to 4 km of axon per mm^3^ gray matter [24]. Yet, little is known about how reliably thin axons conduct APs, because intracellular recordings are difficult to obtain, extracellular data offers only limited signal resolution and stimulus control, and tiny intracellular volumes limit the resolution of imaging methods. Therefore, we used stochastic models of rodent cortical and squid axons [19] to describe the effects of channel noise on the reliability of AP conduction. We demonstrate, contrary to previous assumptions [9,25], that channel noise in thin axons does significantly affect AP conduction, by altering spike timing on the order of a millisecond over distances of millimeters, and we explain how its various stochastic effects degrade spike trains the further spike trains propagate.

## Results

We used the Modigliani stochastic simulator (see http://www.modigliani.co.uk) to model the behavior of unmyelinated axons using a stochastic version of the standard compartmentalized neuron model [19]. We used cable parameters and ion channel models for two very different axons, the rodent cortical and squid axons which operate at different temperatures (6.3° C and 36° C) [19] (see Methods and Tables S2–S6). Optimized numerical code made the simulator's performance and accuracy (length scales of micrometers, time scales down to nanoseconds) powerful enough to study stochastic axons, yet calculating the following results required several months of computer time on a workstation cluster. Our general procedure was to elucidate channel noise effects on propagating APs by measuring the variability across repeated identical trials.

### Four Stochastic Effects in Spike Trains

**Figure 1:**
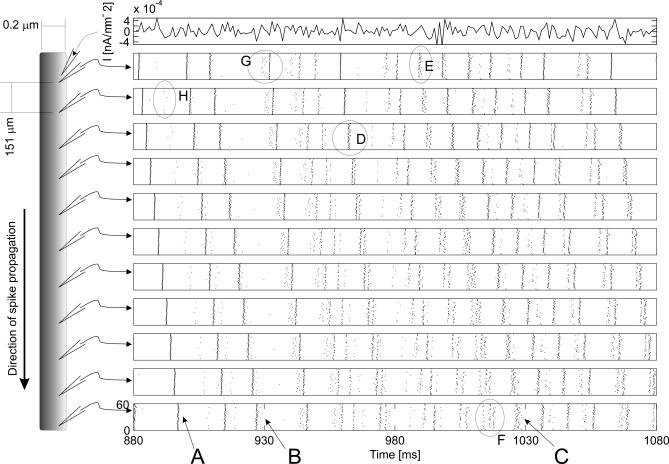
The Stacked Raster Plot Visualizes Traveling APs Produced by Identical Repeated Trials The topmost row shows the stimulus current. Below, each row contains a spike raster plot recorded at equally spaced axonal positions (from the proximal stimulus site at the top to the distal part the axon at the bottom). In each spike raster plot, the precise timing of a spike is marked by a dot on an invisible time line. These time lines are stacked over each other for *N* = 60 repeated trials. The linear shift visible in the overall spike pattern across rows reflects the APs traveling along the axon. Data based on 10-s trials, squid-type axon of 0.2 μm diameter (average diameter of cerebellar parallel fibers). The timing of APs within each set of AP is either unimodal (sets marked by arrows A,B,C), or are markedly multimodal-distributed forming visually distinct groups (set D). In general the timing difference between each group increases as APs travel along the axon (AP sets E and F). APs in a set may be triggered markedly earlier due to differences in the distributions of ion channel states across trials (set G). APs are spontaneously and randomly added (circle H) and in a few cases are even deleted. See text for details.

In a first set of simulations, we explored the effects of channel noise on spike trains. We used an identical “frozen” input stimulus (Figure 1, top row) injected at the proximal end of the axon to elicit spike trains in repeated trials, so that channel noise effects are visible by comparing trials. We can visualize the variability at a given axonal position by plotting the spike arrival times of trials above each other in a spike raster plot. To illustrate how variability changes with propagation, we stacked raster plots for equidistant positions along the axon on top of each other (Figure 1, rows below top row). At the beginning of every trial, the simulation was fully reset—except for the random number generators—such that each trial had identical initial conditions and stimuli, but statistically independent random channel noise events.

In the following we will focus on stochastic phenomena occurring during AP conduction because effects related to AP initiation have been previously described [6,7,9,26]. APs generated by the same stimulus are not precisely aligned across trials, and the misalignment in the AP set grows considerably the further APs propagate. Inspection of the raster plots reveals that APs are added, jittered, split into groups, or deleted as the spike train propagates (Figure 1A–1H). Thus, contrary to previous assumptions [9,25], channel noise has considerable effects on traveling spike trains—well beyond the variability of up to 2 ms introduced by spike generation mechanisms [9,27]. We have identified four distinct stochastic effects of channel noise on AP propagation. To characterize these effects, we call the portion of the input stimulus that triggers an AP its stimulus event. APs that are triggered across trials by the same stimulus event form an AP set.

#### Addition of APs.

The random opening of sufficient numbers of Na channels can trigger a spontaneous AP which does not belong to any stimulus event (see Figure 1, circle H). At the diameter modeled in Figure 1 (0.2 μm), these random, spontaneous APs appear with low rates (1 Hz) and, as previously shown [19], the frequency of spontaneous APs increases exponentially to levels that disrupt communication below a limiting axon diameter of 0.08–0.1 μm.

#### Jitter.

The timing of APs within each set is not unique, but becomes unimodal (Figure 1, arrows A,B,C), or markedly multimodally distributed—forming visually distinct groups of APs (Figure 1, AP sets marked E). At the site of spike generation, all AP sets are unimodally distributed (Figure 1, AP sets marked E and G, or the top raster plot of the AP set marked D). For those stimulus events where the spike time distribution remains unimodal, it is straightforward to quantify the spike train jitter at a given position on the axon as the standard deviation (SD) of spike timing in each AP set, averaged across all AP sets. In general we find that jitter increases with travel distance, so that the spike time distribution of an AP set becomes broader (cf. Figure 1 raster plots second from top with bottom). Jitter increases in two ways, continuously by a gradual amount, and infrequently by sudden discrete jumps (discussed in detail below). For the 0.2 μm–diameter axon shown in Figure 1, the AP initiation at the proximal end of the axon has on average an SD of 0.38 ms, matching values reported in stochastic membrane patches [9]. Jitter then increased over relatively short distances as the spike train propagated, such that at 2 mm distance from the initiation site, the jitter increased to about 0.6 ms SD. This jitter implies that postsynaptic coincidence detection windows cannot be more precise than 2–3 ms at this distance.

#### Spike pattern–dependent splitting (multimodality of jitter).

As the spike train propagates, an AP set can split into distinct groups separated by several milliseconds (cf. spike rasters in Figure 1 AP sets marked by D and E and AP sets marked A–C). Splitting does not occur for all stimulus events, but only for some. In fact, splitting into groups was the only stochastic effect that was not observed in a separate set of simulations (see below), where a single AP was triggered per trial. Therefore, splitting must depend on the temporal pattern of the spike train prior to the stimulus event. In general, the timing difference between each group increases as APs travel along the axon, suggesting that APs in the late groups continue to travel more slowly than APs in the early groups (see Figure 1, splitting into groups of AP sets marked D and F). The SD of spike timing within a group grows in a comparable way to the jitter of a unimodal distributed AP set; splitting is therefore a separate stochastic effect from jitter.

#### Failures.

Channel noise causes very few conduction failures (<1%), even for 0.1 μm–diameter axons, which implies that the safety factor of AP conduction is little affected by channel noise down to the smallest known axons.

All of these stochastic effects must be attributed to ion channel stochasticity because channel noise is the only source of variability and the effects do not occur in the corresponding deterministic axon models given the same stimulus. We can quantify the extent to which these four stochastic effects degrade spike trains, by measuring the information rate conveyed by the spike train along the axon. For the 0.2-μm diameter axon depicted in Figure 1, the information rate (measured following [28], bin widths of 1,2,3 ms) revealed a drop from about 3.5 bits/spike at the site of spike generation to about 2.6 bits/spike 2 mm down the axon. Thus, synapses at this more distal axon position could at most receive 73% of the information available at the site of spike initiation.

### Occurrence of the Four Stochastic Effects Is Insensitive to Axon Model and Parameters

The four stochastic effects are relatively insensitive to model parameters and channel kinetics, since they occur in both squid axon (0.1–0.5-μm diameter; see also Table S1) and rodent axon (0.1–0.4 μm diameter; see also Table S2), which operate with different types of ion channel at very different temperatures, 6.3 °C and 37 °C. In both types of axon, varying membrane parameters (axoplasmic resistance ±50%, membrane resistance ±50%, resting potential ±5.5 mV, temperature ±50%) and ion channel parameters (Na channel density ±50%, Na single channel conductance ±50%, K channel density ±50%, K single channel conductance ±50%) results in either all four stochastic effects being still observed, or the axon becoming so noisy that it is unsuitable for communication, or the axon failing to produce APs. The relative insensitivity of the four stochastic effects to many of these parameters suggests that the effects are linked in a rather general way to the action of channel noise on AP propagation, so that they all become significant in thin diameter axons.

The magnitude or frequency of occurrence of the four stochastic effects increases as axon diameter decreases. AP conduction is sensitive to channel noise because the relative size of channel fluctuations with respect to the number of channels involved grows quickly as diameter 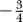 (see Methods for derivation). This relationship implies that, as axon diameter decreases, the number of channels straying from their average behavior becomes as large as the average number of open channels. This always occurred above or at the observed lower limit to axon diameter 0.08–0.1 μm [19].

Having described the four stochastic effects that occur during AP conduction, we consider how these effects are caused by channel noise at a mechanistic level. We previously described in detail [19] how the first of the four effects, the addition of spontaneous APs, is caused by random, prolonged opening of individual Na channels. Therefore, we next address the cause of the two forms of jitter, gradual conduction velocity fluctuations and sudden jumps. Thereafter we consider the third and fourth effects: APs splitting into groups and failures/deletions.

### Stochastic Mechanisms Causing Jitter: Gradual Conduction Velocity Fluctuations

**Figure 2:**
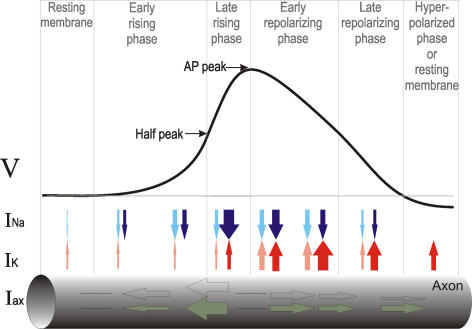
Diagrammatic Representation of a Traveling AP on the Axon Stacked over each other, the membrane potential wave form of the AP (V) along the axon, axial currents flowing along the axon (*I_axial_*, green), Na (*I_Na_*, blue), and K currents (*I_K_*, red), and the representation of the axon itself. Axial, Na, and K currents are denoted by green, red, and blue arrows scaled to represent the relative size of the current in the various AP phases. Hollow and light-shaded arrows denote the size of the current fluctuations relative to the average currents. The AP wave form is subdivided into six phases: resting membrane, early rising phase, late rising phase, early repolarizing phase, late repolarizing phase, and an optional hyperpolarized phase (as some types of axons do not hyperpolarize). See text for details.

To understand how channel noise can gradually vary the speed at which APs travel down axons, we will briefly consider how the AP propagates (see overview in Figure 2). The regenerative cycle of AP propagation begins with a local, suprathreshold membrane depolarization opening Na channels, which produce an inward current that further amplifies the depolarization in a positive feedback loop (Figure 2, rising phases). The resulting potential difference between the local depolarization of the axon and the resting membrane further ahead produces an axial current flowing down the membrane potential gradient and ahead of the wavefront peak (Figure 2, black arrows). This axial current depolarizes the resting membrane, again triggering the opening of Na channels, and so the cycle begins anew. Any variability occurring after and behind the AP peak will have little effect on propagation speed, because the repolarizing membrane cannot drive currents forward (Figure 2, repolarizing phase). Thus, propagation depends on the forward-flowing axial current produced by the rising AP.

**Figure 3:**
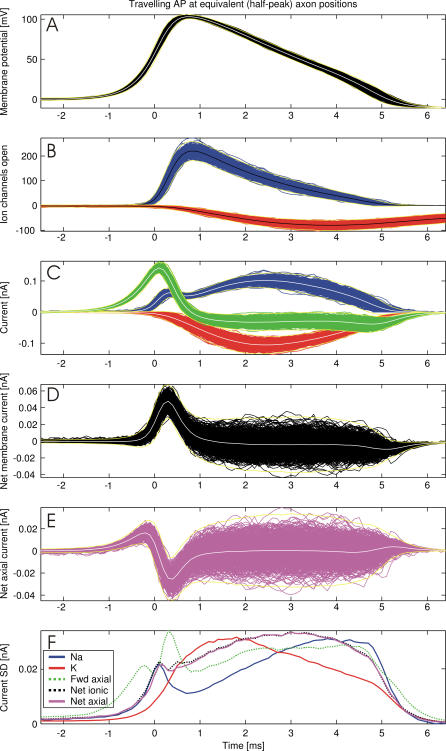
Variability of the Propagating AP Aligned at Half-Peak Membrane Potential (*t* = 0 ms, Data for 0.3 μm Diameter Axon, White/Black Curves Are Arithmetic Averages and Yellow Curves are 3 × SD Envelopes, *N* = 673) (A) Membrane potential wave forms. (B) Open Na (blue) and K (red) channels. (C) Ionic currents flowing through the membrane (Na current, blue; K current, red) and axial current (green). (D) Net membrane current calculated as the sum of Na, K, and leak currents. (E) Net axial current is the sum of inflowing and outflowing axial currents, describing where axial current is produced (negative values) or consumed (positive values). (F) SDs of the currents in (C–E), color coding as above. Data shown are the overlayed traces of a single AP propagating along 711 axonal compartments. See text for details.

To measure how channel noise affects axial current, one has to track the quantities affected by channel noise as the AP moves along the axon. To this end we triggered single APs in both axon models at various diameters and recorded the time series of the membrane potential and the number of open channels in narrowly spaced axon sections (spacing was 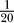 of the axon's length constant, all diameter and parameter variations kept this spacing <20 μm). The time series recorded at each position are superimposed, after having been aligned at the instant when the membrane potential crosses its half AP peak value. This procedure directly displays the relevant quantities and their variability at corresponding phases of the traveling AP waveform (cf. overview Figures 2 and 3 showing data for a 0.3-μm diameter squid axon).

### Channel Activity and Ionic Currents during the AP

The majority of Na channels open just before the membrane potential reaches the AP peak (Figure 3A and 3B at 0.3 ms), while most K channels open when the AP is already repolarizing (Figure 3A and 3B at 2.5 ms). In contrast, Na and K currents reach their maximum earlier (in Figure 3C at 0.3 ms and 2.5 ms) due to the larger electromotive forces. Maximum forward axial current is produced close to half-peak (in Figure 3C between 0 ms and 0.15 ms) because the membrane potential gradient is steepest here. Yet, in marked contrast to AP initiation, during AP propagation the ionic (Na and K) currents trail the early rising phase of the AP (in Figure 3C between −1 ms to 0 ms). This is because AP propagation is driven by axial currents depolarizing the membrane ahead, which then opens Na channels. Therefore, the rising AP phase extends as far ahead as the forward axial current (in Figure 3C at −1 ms).

The net current flowing across the membrane peaks in the late rising phase as the AP peak is reached (in Figure 3D between 0.2 ms and 0.4 ms). The net axial current (the difference between axial current flowing on or off the membrane capacitance) peaks here, too (in Figure 3E between 0.2 ms and 0.4 ms), because the axial current in this region is sustained by local Na currents causing most of the depolarization. The net axial current goes from negative to positive close to half-peak, as the late rising phase (between half-peak and peak) contributes more axial current than it receives (due to the rapid increase in the rate of depolarization caused by Na^+^ channels), while the early rising phase (between resting axon and half-peak) behaves vice versa (as it consumes the axial current by depolarizing the membrane up to threshold; cf. Figure 2).

### Fluctuations in Channel Numbers and Variability of Currents during the AP

The variability of the currents at corresponding points in time (here measured as SD) are also the fluctuations at different axonal locations encountered by the AP waveform as it travels down the axon. In Figure 2 these fluctuating currents are indicated by hollow or light-shaded arrows, scaled in relation to the average currents. Na and the forward flowing axial currents are more variable in the early rising phase (respectively, −0.5 to 0 ms and −1.3 to 0.3 ms in Figure 3E). The SD of the current fluctuations is comparable to the size of the currents in this phase, highlighting the fact that in thin axons channel fluctuations will become as large as the maximum number of open channels. A simple analytical approximation (see Methods), shows that the relative size of the fluctuations goes as 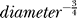 ; thus, channel fluctuations are bound to grow as large as the average number of open channels, as axon diameter decreases. This relationship is a general consequence of the cable properties of axons and the binomial nature of open channel fluctuations; it is independent of the axon's membrane or ion channel properties.

The net axial current in the early rising phase (0.004 ± 0.002 nA at *t* = −1 ms in Figure 3C) is a small fraction of the net axial currents maxima (0.02 ± 0.018 nA in Figure 3C), and small in comparison with the axon's rheobase (0.03 nA for axon in Figure 3C)—the minimum stimulus current required to trigger an AP. Note, that almost all fluctuations are within a 3 × SD envelope (yellow lines in Figure 3), showing that very large fluctuations are unlikely to occur (<1% of the traces for Figure 3). As K channels are predominantly closed, the net membrane and axial current variability must result from Na channel noise (cf. matching SD curves between −1 and 0 ms in Figure 3E). These fluctuations result from Na channels opening with delay or failing to open, as well as Na channels that start inactivating prematurely. Thus, both the rising phase of the wavefront and the resulting axial current are determined by Na channels and their fluctuations.

The largest absolute variability in the number of open K channels occurs between the AP peak and the late repolarizing phase. K currents have little effect on the axial current contributing to propagation, but contribute to large fluctuations in the rate of repolarization. This results in variability of the AP waveform width and height which by the time an AP reaches a synapse can significantly vary the Ca^++^ signal driving synaptic transmission (unpublished results).

We show that the profile of fluctuations in the currents (illustrated in Figures S3–S6) is maintained over a 10-fold range in axon diameter. This observation emphasizes that the profile of fluctuations is inherent to the AP voltage profile and thus the AP mechanism itself. Thus, we are confident that our principal conclusion is robust; namely, that Na channel noise in the early rising phase AP (around AP threshold depolarizations) varies the speed of membrane depolarization to the AP peak, and in turn causes fluctuations in the axial current driving the AP.

### Variations in Current Are Linked to Variations in Propagation Velocity

**Figure 4:**
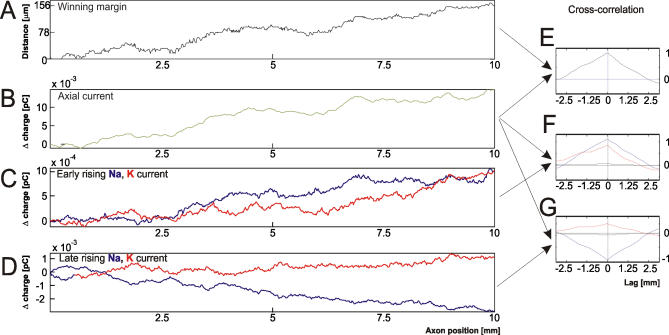
Comparison between the Fastest and the Slowest AP Out of 250 Single AP Trials Data shown for a 0.3 μm diameter axon (average diameter of cortical axon collaterals). (A) Margin between the faster and slower AP wavefront after propagating the same amount of time. (B) Difference in the charge supplied by Na (blue) and K (red) channels between the foot of the AP and half-peak. (C) Difference in the charge supplied by Na (blue) and K (red) channel between half-peak and peak of the AP wavefront. (D) Difference of the axial charge generated at identical points in time. (E–G) Show the unbiased cross-correlations between the quantities depicted in (A–D), as indicated by the connecting arrows and color coding.

To establish that fluctuations in axial current, and hence early Na current, are related to variations in propagation velocity, we analyzed 250 identical repeated trials where a single AP was triggered. We repeated these blocks of at least 250 trials on both the rodent cortical and the squid axon models with diameters ranging from 0.08 μm to 1 μm. Unlike the first spike train protocol, splitting was not observed, while additions, jitter (gradual and jump-like), and failures were observed. For every axon diameter studied, the fastest and the slowest trials were selected and the winning margin (the distance gained by the AP in the fast trial over the AP in the slow trial after propagating for the same amount of time) was tracked. This allows one to compare if instantaneous differences in channel behavior between the faster and slower trials translate into instantaneous changes in propagation speed (Figure 4A). Note that, since we are studying gradual conduction velocity changes here, we chose APs without jump-like conduction events (see next section).

We present the results and illustrate them for a 0.3-μm diameter axon in Figure 4. The excess axial current produced by the faster AP was strongly correlated with the distance gained by the faster AP over the slower AP—the winning margin (Figure 4E). The excess Na^+^ influx (measured in units of Na^+^ charge) produced under the early rising phase (red curve in Figure 4C) and the excess axial current (Figure 4B) in the faster AP correlated well with each other (red curve Figure 4F). Note that this correlation was weaker in the late rising phase. In contrast to the early rising phase, K currents in the late rising phase (green curve in Figure 4D) are anticorrelated with the axial current (green curves in Figure 4G). Furthermore, Na and K currents in both rising phases show very weak correlations with each other (black curves in Figure 4F and 4G).

**Figure 5:**
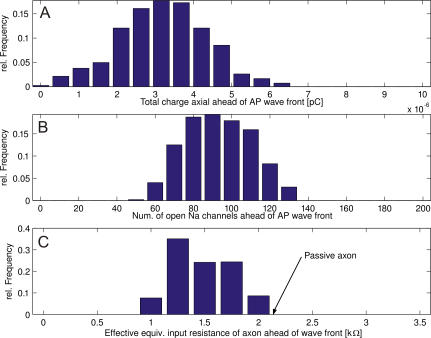
Variability Experienced by Propagating APs (Data for 0.3 μm Diameter Axon, *N* = 671) (A) Axial charge generated by incoming AP. (B) Number of open Na channels under the rising foot of the AP. (C) Input resistance of the resting axon as experienced by the incoming AP wavefront. Arrow indicates the axonal input resistance when all voltage gates are closed.

This chain of evidence shows that Na channel fluctuations in the early rising phase (cf. Figure 5A) determine the variability in axial current (Figure 5B). The axon's input resistance also affects the speed by which the axial current of an incoming AP depolarizes the resting membrane ahead of it. The resting axon's input resistance fluctuated considerably as K (and very few Na) channels spontaneously opened and closed (Figure 5C, 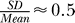 ). This is because the resistance of an individual channel is within an order of magnitude of the axonal input resistance at diameters below 0.5 μm (single channel conductance of known (axonal) voltage-gated Na and K channels: average ≈20 pS; range 10–50 pS [29]). Thus, channel noise produces conduction speed variability, because the AP is driven by events occurring well ahead of the AP peak, where the number of ion channels involved is small and their relative variation from the mean large.

### Stochastic Mechanisms Causing Jitter: Jump-Like Propagation by Stochastic Microsaltatory Conduction

**Figure 6:**
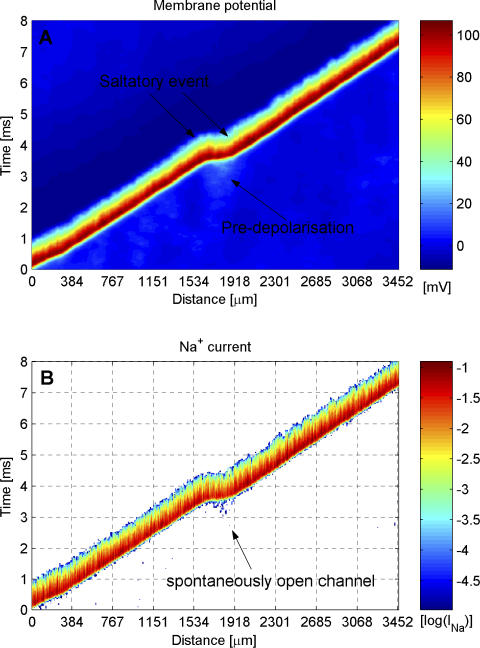
Visualization of a Stochastic Microsaltatory Conduction Event in a 0.1 μm Diameter Squid Axon A continuously propagating AP wavefront (note the jitter in propagation speed) suddenly jumps from position *x* = 1,550 μm to *x* = 1,900 μm. Displays a space-time plot of the membrane potential on the axon (A) and the corresponding space time plot of the transmembrane Na current (B). The regions where no Na current was flowing are white, making ionic currents from spontaneously open channels clearly visible.

Some APs travel faster than expected of a continuously moving wavefront. We called this effect stochastic microsaltatory conduction. This is due to collaborative effects among Na channels in the resting axon ahead of an AP (see Figure 6), where the random opening of nearby Na^+^ channels pre-depolarizes a region of the membrane by a few millivolts. The axial current from an incoming AP is then sufficient to immediately reach the AP threshold, skipping the comparatively slow increase of the early rising phase toward threshold. The AP wavefront leaps several hundred micrometers ahead to the pre-depolarized region, and the spike time at a given position is shifted by a few milliseconds. This stochastic microsaltatory conduction resembles saltatory conduction between morphologically specialized Nodes of Ranvier in myelinated nerve fibers, but it is produced by the stochastic behavior of individual channels embedded in an axon of uniform morphology. Thus, saltation occurs at random positions and times and adds considerable variability across trials. We observed jump-like propagation in simulations of both spike trains and single APs.

We investigated a previously proposed mechanism of microsaltatory conduction [30], where at very low Na channel densities (<1μm^−2^) APs were thought to jump from Na channel to Na channel in 0.1-μm diameter axons. We found that this proposed mechanism cannot work, because at these very low channel densities the stochastic behavior of the single Na channels causes propagation to fail. Na channel densities above 2–4 μm^−2^ were required for a 0.1-μm axon to support conduction, at which point the number of channels sustaining an AP is large enough to conduct APs continuously (see Figure S2).

### Splitting Is Caused by Memory Retained in Ion Channel Populations

Splitting is caused by gradual conduction fluctuations and jump-like events acting together in a structured manner, such as to systematically accelerate or decelerate APs within an AP set as it propagates along the axon. Were the occurrence of these jitter effects not structured but entirely random, then their summed effect would have to converge toward a unimodal spike time distribution by the law of large numbers.

The opposite is the case, because the modes of the spike time distributions become increasingly separated (cf. Figure 1: Figure 1D and bottom raster plot). Note also that at the site of spike initiation the distribution is unimodal. We can make the splitting effect disappear (no multimodal spike time distributions) by modifying the Markov model of our ion channels. To achieve this, the transitions among the closed/inactivated states of ion channels have to be made deterministic, while transitions between the open state and the adjacent closed or inactivated states remain stochastic (unpublished data; this corresponds to a simplified channel model previously used [25]). The jitter in this simplified model matches simulations where deterministic Hodgkin–Huxley ionic conductance models of gating were made noisy by adding white noise ionic currents on top of the deterministic ionic current [25]. We conclude that it is essential to account for the stochastic transition between the different closed channel states to account for splitting and grouping.

The observation that the splitting effect disappears in the simplified model implies that ion channels retain information about previous activity in such a way that spike time reliability is linked to the pattern of spikes.

What variables could be responsible for these differences in reliability? We can eliminate the stimulus current—it is identical from trial to trial and from the membrane potential—because it is reset during the refractory phase of the AP in a stereotyped fashion (note in Figure 5 that when APs are aligned there is negligible variability when the refractory phase begins; *t* > 5.5 ms, SD < 1mV). The only free variables left are the distributions of non-open states across the populations of ion channels (i.e., the only variables that are not unconditionally set by the membrane potential). These distributions can vary because they are conditionally dependent on membrane potential. Note that in the traditional Hodgkin–Huxley model of ionic conductances, the membrane potential directly sets the gating particle states. Thus, the state of a channel prior to a membrane potential change is ignored. This is not the case if one accounts for the stochastic nature of transitions between discrete conformational states of the channel protein, as in the biophysically realistic multistate Markov models [29]. In the detailed channel description considered here, the membrane potential sets only the conditional probability of a state transition. This allows fluctuations in populations of ion channels to remain correlated for many milliseconds, persistently deviating from their mean behavior (e.g., the mean excess ion charges in Figure 4B and 4C are far from zero and persistent for many milliseconds), and this results in the splitting effect. These correlations are much longer than the expected time of a single ion channel to remain in its state (single channel open state correlations are on the order of typically <0.1 ms [29]). This also explains why stochastic microsaltatory conduction, which relies on groups of several spontaneously opening Na channels to overlap in their opening time long enough to facilitate conduction, becomes so unlikely in the simplified model that it was not observed.

### Failure of APs

We found that APs are unlikely to fail to propagate due to channel noise. In simulations where a single AP per trial propagated down an axon, in both models and at diameters ranging from 0.5 μm down to 0.08 μm, less than 1% of APs failed to reach the axon terminal (*N* = 250 per model and diameter pair). Failure is unlikely because the AP mechanism requires a large, inactivating Na conductance to depolarize the membrane and a smaller K conductance to reversibly oppose this effect. The high open probability above the AP threshold voltage and the large depolarizing current of a single Na channel produces a strong positive feedback once the AP has been triggered. If Na channel densities are sufficiently high (≥4 μm^−2^, shown above), this will ensure that sufficient numbers of Na channels sustain the AP even when individual Na channels remain closed. For conduction failure then to occur would require large numbers of Na^+^ channels to fail to open, which is many orders of magnitude more unlikely than a single channel failing to open. The failure probability *p* for single Na channels above the AP threshold is small (*p* < 0.5) and, hence, the failure probability of *n* channels is much smaller and proportional to *p^n^* = 0.5 [29]. For analogous reasons, very large numbers of K channels opening spontaneously and counteracting the depolarizing Na current upstroke of the late rising AP phase are similarly unlikely, thus propagation seldom failed.

## Discussion

While it has been established that channel noise causes spike-initiation variability, and generates spontaneous APs [19], its effects on spike propagation variability have not been rigorously analyzed before. We have shown here that channel noise adds previously unreported stochastic, history-dependent effects which corrupt spike trains the further they propagate in thin axons (<0.5-μm diameter). Because channel noise perturbs APs during their entire journey along the axon, propagation variability will exceed spike initiation variability at a distance on the order of millimeters, that is, shorter in narrower axons. In axons comparable to parallel fibers (0.2 μm diameter), spike time jitter increased to 0.6 ms SD over a distance of 2 mm, which implies that postsynaptic coincidence detection windows cannot be more precise than 2–3 ms. The information rate dropped linearly over this distance by more than 25%.

Conduction variability is a direct, inescapable consequence of internal noise in the AP signaling system and will emerge whenever the input resistance of axons becomes so large that small numbers of ion channels can support AP conduction. This is because, counterintuitively, only a very small proportion of Na channels contribute to the advancing subthreshold “foot” of the AP. Note that the input resistance of thin axons comes close to, or is of the same order as the resistance of, a single open ion channel. Consequently, as we previously demonstrated with spontaneous APs [19], channel noise effects are both significant in thin axons and robust, i.e., they are resistant to biologically plausible changes in biophysical parameters such as channel densities, the use of different types of channel (e.g., the squid channels versus rodent cortical channels), leak conductance, and temperature (see Figures 2–5; and Supplementary Information S1 in [19]). Channel-noise effects become more severe as diameter decreases so as to render axons below 0.08–0.1 μm diameter inoperable for communication. Thus, channel noise is bound to affect conduction in thin axons below 0.5 μm diameter, unless their ion channels and membrane properties are very different from known axons. Therefore, axons above 0.5 μm diameter are going to be reliable. For the same reasons, dendrites that support backpropagating APs and dendritic AP initiation are typically thicker then 0.5 μm and were found to reliably conduct APs [13]. Our results also show that by using more realistic multistate models to account for the stochastic behavior of ion channels, one observes new results that cannot be captured by classic deterministic models, even if they are enhanced by Gaussian approximations of channel noise.

### Spike Time Jitter and Ion Channel Gating

Axonal spike time jitter has previously been measured in myelinated cat and frog axons of several μm diameter and was on the order of 0.01 ms [31,32]. The jitter we report here in stochastic simulations of unmyelinated axons between 0.1–0.5 μm diameter was on the order of 0.1–1 ms SD over distances of millimeters. In contrast, previous stochastic simulations of axons [25] underestimated channel-noise effects in axons (of comparable diameter) by several orders of magnitude (spike time variability SD 0.001–0.01 ms). Those pioneering simulations implemented a simplified kinetic channel model which ignored stochastic transitions between closed ion channel states, i.e., only transitions between the open and the nearest closed states were stochastic, all other state transitions were deterministic. It is not surprising that simulations with simplified channel kinetics matched the spike time jitter of previous simulations that used deterministic conductance models with additive Gaussian current noise [25]. A Gaussian channel noise model generates uncorrelated noise, and cannot therefore retain a memory of previous activity in the distribution of states across a population of ion channels. The simulations presented here use detailed kinetic models of channels that establish and preserve correlations in space and time, and this allows APs to travel persistently faster or slower than average (splitting, Figure 1) or to jump relatively large distances (Figure 6, stochastic microsaltatory conduction). Furthermore, the earlier simulations [25] used spatial compartments and time steps that were 10–100 times coarser than ours (probably to overcome severe computational constraints). We used our model to verify that this coarser discretization averages out noise (unpublished data).

The unsuitable Gaussian channel noise model corresponds to a Langevin-type approximation of the Markov process of channel gating [33]. Langevin approximations break down in iso-potential membrane patches with fewer than about 800 channels [33], but their limitations have not been assessed in non-iso potential models of neurons since the number of channels involved dynamically varies with distance, membrane potential, and conductance. Based on our simulations, we conclude that the accuracy of Langevin-type approximations breaks down in axons below 1 μm diameter. Note that for typical channel densities and assuming that the average membrane area affected by the AP corresponds to an axon cylinder about a length constant long, the breakdown limit corresponds to at least an order of magnitude more channels than in membrane patches. Thus, Langevin-type approximations of channel noise are likely to break down in smaller arborized neurons, because they ignore both spatial and temporal correlations.

An important study on stochastic simulations of membrane patches used realistic channel kinetics and showed that spike initiation has high temporal precision when the size of ionic current fluctuations near the AP threshold are small compared with the injected stimulus current [9]. Thus weaker stimuli will produce more unreliable spiking in agreement with experimental data [27]. These results were extrapolated to axons, where the axial current driving the AP was regarded as strong driving input, and, hence, it was inferred that APs should propagate very reliably in axons [9]. However, the spatial spread of membrane potential follows different input–response relationships than in point-like iso-potential membrane patches [34]. In fact, the current driving the AP ahead is smaller than the minimum stimulus current required to trigger an AP in the axon (Rheobase); in our simulations it is one to two orders of magnitude smaller. Consequently, contrary to previous suggestions [9], the driving axial current is a weak input that is susceptible to channel noise, and we find that conduction velocity fluctuates significantly, because the small number of channels involved in driving the AP, when the membrane is between resting potential and AP threshold, are subject to large fluctuations.

### Stochastic Microsaltatory Conduction

Our simulations reveal a second source of jitter in thin axons, stochastic microsaltatory conduction, whereby the AP jumps well ahead of the advancing wavefront to a cluster of spontaneously open Na channels. Thus, this cluster of spontaneously open channels acts like a Node of Ranvier. Our simulations revealed that this collaborative channel effect occurs only in thin axons with high Na channel densities. In contrast, we show that the ingenious suggestion that thin axons could be dotted with individual Na channels—each acting like a Node of Ranvier [30] —produces axons that are too unreliable to sustain AP conduction.

### Axonal Memory Residing in Populations of Multistate Ion Channels

Spike trains are subject to stochastic effects which depend on the history of previous axonal activity (splitting into groups), while single APs did not. Thus, information about previous activity must be retained in the state distribution of ion channels. This effect should be measurable in axons below 0.5 μm diameter. Axonal memory effects have been previously measured in axons [35], where selective conduction of APs was, for example, dependent on inactivation of a K channel [36]. Here, we show that the timing reliability of APs can become dependent on the axon's activity history. This is a general property of any voltage-gated ion channel with multiple states. This property could explain evidence showing stimulus-dependent spike time reliability [37], by channel noise affecting different patterns of spikes to a differing degree. Because spike time reliability depends on the temporal pattern of spikes (splitting effect), dendritic and somatic mechanisms of AP initiation could be tuned to generate spike patterns that propagate especially reliably. Such reliable spike patterns could involve bursts, because our findings suggest that the first spike of a burst will condition channel noise effects for subsequent spikes, such that the interspike interval between the second and subsequent spikes of the burst will be more reliable than the interval between the first and the second.

### Conduction Safety in the Presence of Channel Noise

Although channel noise provides an obvious means for APs to fail, our results show that conduction failures are rare (1% in axons of the smallest known diameter and thus the noisiest axons). More often than not, channel noise has the opposite effect of promoting AP generation (e.g., additional spontaneous APs, stochastic microsaltatory conduction). Our findings suggest that conduction safety will be as high in thin (0.2-μm diameter [38]) myelinated axons as in unmyelinated axons of the same diameter, because with very high Na^+^ channel densities and very low K^+^ channel densities at the Nodes of Ranvier, the strong positive feedback of AP regeneration is even less likely to be overcome by channel noise. We are currently working on modeling channel noise effects in myelinated axons, as many long-range connections, such as cortico–thalamic connections, are often partially myelinated, unlike the unmyelinated axons discussed here.

Experimentally, thin pyramidal cell axons were found to conduct APs reliably [39,40]. Reliable conduction was observed in dissociated cells of cortex and hippocampus, where intrinsic properties such as channel noise are conserved, but effects depending on extracellular or network mechanisms are lost. However, the same cell types displayed propagation failures in nondissociated tissues [41]. In fact, several neuronal mechanisms that purposely produce conduction failure are known, acting through membrane hyperpolarization, shunting effects, and spike-frequency–dependent block at branch points (see [42]). Our findings suggest that channel noise cannot account for the AP failures observed in many systems and that other factors must be responsible. We suggest that if propagation failures occur in the healthy nervous system, then these are due to purposely designed mechanisms for presynaptic information processing, which allow the incorporation of local information not available at the site of spike initiation. We note that channel noise effects at branch points and other low-safety factor regions of the axon (e.g., synaptic varicosities) could be used for probabilistic routing or editing of spike trains, and further stochastic simulations are required.

### Experimental Validation and Synaptic Transmission

Testing the predictions of our theory (the five stochastic effects) is very straightforward. If one replicated our stimulation protocol on axons of similar diameter, one would only have to look at the raster plot of paired recordings along an axon or at somatic and postsynaptic activity, and these should reveal the effects described in our simulations. In fact, we suspect that existing data on paired cell recordings could straightforwardly reveal some of these novel effects, if reanalyzed appropriately, i.e., by not discarding “abnormal” trials that could well have been caused by axonal channel noise effects (e.g., where a spontaneous AP triggered synaptic response or where the postsynaptic response preceded the presynaptic stimulus). A practical problem in relating existing experimental data to our results is that the diameters of the axons are unknown, often because the necessary electron microscopy data was not, or cannot be, obtained in some preparations.

A straightforward experimental system would be to cultivate individual pyramidal cells on a dish, where they can form individual long-range axons and have them grow over micro-electrode substrates (Hugh Robinson, personal communication). The occurrence of the stochastic effects could be facilitated, such that they occur in larger diameter axons. A counterintuitive and easily testable prediction is that decreasing temperature will increase channel noise effects on APs, e.g., as spontaneously open channels would stay open longer and increase the amount of charge delivered to the membrane [19]. Furthermore, decreasing the numbers of available Na channels should (e.g., by pharmacological blocking a proportion of them) increase propagation speed jitter, while increasing Na channel numbers and decreasing K channel numbers should facilitate the appearance of spontaneous APs as well as saltatory conduction events.

Spike time reliability is bound to decrease the farther the AP travels, thus long-range communication is in this respect noisier than short range communication, given the same axon diameter. Nonetheless, axonal channel noise may have an effect on information transmission in short-range synaptic connections, because the AP wave form is perturbed by channel noise (cf. Figure 3). The AP wave form in turn determines the Ca signal at the synapse, which controls vesicle release. Thus, as will be shown elsewhere, short-range synaptic connections may experience trial-to-trial variability in release probability and postsynaptic responses, due to fluctuations of the driving AP wave form. Furthermore, synaptic channel noise at presynaptic Ca channels and postsynaptic receptor channels may produce spontaneous postsynaptic potentials and further increase trial-to-trial transmission variability.

### Robustness of Noise Effects

Our extensive parameter variations demonstrate that the precise means by which channels generate noise has a limited effect (see Tables S1 and S2). Thus, the findings we present here are rather general. Indeed, the key finding of our paper is that the specific details of ion channels, kinetics, or the gating model are negligible compared with the fact that channel gating per se is stochastic.

The novel noise effects that we report depend on six basic factors that are fundamental to the mechanism of AP propagation, namely; a) Na channels supporting the AP have to open earlier than the opposing K channels, b) Na channels have to inactivate to allow for repetitive unidirectional signaling, c) channels are discrete all-or-none conductances, d) the fact that channels gate stochastically, e) the AP is locally initiated by a relatively small number of Na channels, due to the way that depolarization spreads in cables, and f) the single channel conductance approaches the input conductance of the membrane as axons become thinner. These basic factors suffice to explain the observed effects. Furthermore, the multistate nature of channels produces the novel effect of activity “memory” across a population of ion channels that can last an order of magnitude longer than an individual ion channel time constant.

We have tested our axon simulations with several Na channel models that differ with respect to activation/deactivation/inactivation kinetics, and the overall gating scheme. We modeled squid axon using a Na channel model where among other things, activation and inactivation are not independent [43]. We observed the same stochastic effects as in the case of the standard squid channel model. We also simulated the pyramidal cell axon using a model of hippocampal Na channels [44], which has more states and kinetic functions.

The more recently proposed models for cortical Na channels [45,46] have not been included in our study. This is not only because they are controversial (see discussion in Text S1 and also [47]), but also because they increase the rate at which the membrane potential changes just above threshold. Therefore, they will produce a greater decrease in spike time reliability[46] than the models used here. Thus, our data suggests an upper bound to the reliability of pyramidal cell axons.

We conclude that, as in our previous work [19] where we showed that channel noise sets a lower limit to axon diameter that is remarkably insensitive to the specific details of the channel model, all novel stochastic effects on AP propagation we reported here occur irrespective of the specific channel gating model, at least within plausible limits.

### Conclusion

We have studied the AP in thin axons and have shown that stochastic modeling of individual ion channels captures essential properties and constraints of whole cell behavior. These effects cannot be accounted for by deterministic (e.g., jitter) or approximative (Langevin-type) stochastic models (stochastic microsaltatory conduction). Conversely, we find that axons (or dendrites) above 0.5 μm diameter will reliably conduct APs, and deterministic modeling of ion channels should suffice for these, unless AP initiation is considered.

Our findings prompt careful experimentalconsideration in thin axons, because typical experimental protocols are not geared to distinguish postsynaptic variability due to synaptic transmission from variability caused by axonal noise (“axonal variability”). Optical methods and extracellular recordings have limited stimulus control and signal resolution; thus intracellular paired-cell recordings, as used to study small synapses innervated by thin axons, would be more suitable. However, the impact of axonal channel noise in circuits may have gone so far unnoticed, because paired-cell measurements which could reveal axonal variability are difficult to obtain, since typical cell-to-cell distances are limited by the microscope's field of view and the probability of finding two monosynaptically connected neurons to about 500 μm—at this distance conduction jitter would be only on the order of 0.1 ms. However, cortico–cortical axon collaterals and cerebellar parallel fibers, which are below 0.5 μm diameter, can extend up to a centimeter away from the soma, suggesting that jitter will limit the minimum width of reliable coincidence detection windows to 5–10 ms at their terminals.

Thin unmyelinated axons are typically used to innervate large numbers of small synapses [48] and are, therefore, associated with and required for the high level of circuit miniaturization observed in our brains [19]. Circuit and axonal miniaturization is beneficial to an organism's evolutionary fitness, because it decreases connection length, mass, volume, and metabolic demand of brains [19,49]. Our findings show that noise poses a limit to miniaturization, because the information capacity of axons will rapidly decrease as they approach the limiting diameter of 0.1 μm—making them both slow and noisy. Behavioral requirements on information processing and neural coding are thus intricately linked to brain anatomy by biophysical constraints such as axonal channel noise and provide a rich area to investigate these evolutionary relevant relationships using computational methods. We have studied the role of internal noise-setting constraints on the design of a typical cell-signaling system [50], the AP. Similar stochastic effects and the general relationships will govern other pulsatile signaling systems that rely on inherently noisy protein switches to generate and propagate signals, such as Ca and cAMP waves.

## Materials and Methods

All simulations were carried out using our Modigliani stochastic simulator (see http://www.modigliani.co.uk [19]) on Linux workstations and a Linux cluster and verified using deterministic simulations using Neuron (see http://neuron.duke.edu). Channel gating was described by discrete state Markov processes, capturing the corresponding ion channels' kinetics from patch clamp experiments. Two different stochastic integration algorithms (Gillespie, Binomial; see [19]) were used and an additional set of deterministic simulations were carried out to cross-verify simulation results. Time step sizes for the Binomial algorithm where 1 μs (results verified using 0.05 μs), while the Gillespie algorithm generated steps as small 1 ns. Spatial discretization was, depending on parameters, between 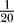 and 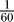 of the axon's length constant (results verified with 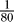 ).

In the first (spike train) protocol, the axon model was simulated for *N* = 60 repeated trials of 10 s each. The unmyelinated axon of 0.2 μm diameter (2 mm long) was modeled using squid axon channels kinetics (Na,GFLN1; K,SqKv1.1; see Table S3) and channel densities following [19]. In this first protocol we use a hybrid model of high resistance mammalian membrane (e.g., [51] report values of up to 70,000 Ωcm^2^ for cortical interneurons) with squid-like ion channels. Cable properties were here *R_a_* = 70 Ωcm and *R_m_* = 20,000 Ωcm^2^. Axon length was kept short to reduce computing times, yet the length and number of trials required several weeks to complete for one parameter set. Spike trains were generated by stimulation with a zero-mean white noise current (SD = 0.01 nA, 1-kHz corner frequency) injected at the proximal end compartment. After each trial, the simulator reset to identical initial conditions (only the state of the random number generators were preserved).

In the second (single AP) protocol, an individual AP was triggered by a rectangular input pulse and propagated down the axon. Two types of axon models where used, a squid axon model (channels as above and squid membrane parameters; for details see Table S4 and Figure S1) and a cortical pyramidal cell axon collateral model based on data from rodent cortex [19] (for details see Table S5). The respective cortical rodent ion channels modeled were Nav 1.2 and Kv1.1/3. Each trial simulation was run until after the AP reached the distal end of the axon, and as before the simulator was reset after each trail. Axons of diameter 0.08, 0.1, 0.2, 0.3, and 0.5 μm (1 cm long) and 1 μm diameter axon (2 cm long) were stimulated in a single spike per trial framework (*N* = 250 trials). Parameter variations where carried out by modifying a single parameter while keeping all other parameters constant.

After visual inspection of the data, APs were detected by a threshold discriminator detecting half-maximum AP height and their waveforms aligned at their threshold crossing time to construct the raster plots and current traces. Spike timing was quantified by first calculating the SD of the spike timings within each event and then averaging across all events. Spike train information rates were measured and linearly extrapolated according to [28].

### Scaling Relationship between Channel Fluctuations and Diameter *d*.

The total number of channels, *n,* involved in the rising phase of the AP is proportional to the membrane area affected by its depolarization. This area, a cylindrical section of membrane, is proportional to the circumference (∝*d*) of the axon, times the electrotonic length of the axon (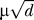 ). Thus, keeping channel density constant, the number of affected channels goes as the 3/2-power of diameter (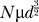 ). The number of open channels, *N_O_,* is *N* times the voltage- and history-dependent channel open probability *p*. Thus, the binomially distributed SD of *N_O_* is 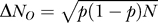 . The ratio between the size of the fluctuations and the total number of channels scales with diameter is 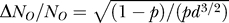 . Thus, the relative effect of channel fluctuations grows quickly as 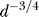 as diameter decreases. Note, that *p* is generally low. At half-peak membrane depolarization *N_O_* is a fraction of *N_O_* at peak. Even at peak, *N_O_* is typically between 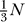 to 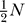 . Thus, the low open probabilities during the rising phase of the AP contribute to any diameter-dependent channel noise effects. The overall relationship is given by the basic biophysics of AP signaling and is independent of the specific axon parameter or channel kinetics.

## Supporting Information

Figure S1 Markov State Transition Scheme of the Patlak Na^+^ Channel Model (52 KB DOC)

Figure S2The Plots Show Superimposed Voltage Traces of Squid Axons with Different Channel Densities(Top) Na^+^ channel density was set to 2 mm^2^ (top) and 4 mm^2^ (bottom). The K^+^ channel densities were adjusted to keep the 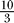 Na_+_/K_+_ density ratio constant. Each row plot contains the superimposed voltage traces *V*(*x*,*t*) (in units of millivolts) for 20 repeated trials. Each row corresponds to a recording position *x* along a 0.1 μm diameter axon of 1 mm length. APs are traveling from left to right and were triggered at *t* = 0 ms. Note that as the trials are superimposed, the variability in spike triggering is clearly visible, as well as is some back-propagating SAPs.
(425 KB TIF)

Figure S3 Variability of the Propagating AP Aligned at Half-Peak Membrane Potential (*t* = 0 ms, Data for 0.2 μm Diameter Axon, White/Black Curves Are Arithmetic Averages and Yellow Curves Are 3 × SD Envelopes, *N* = 673) (A) Membrane potential wave forms.
(B) Open Na (blue) and K (red) channels.
(C) Ionic currents flowing through the membrane (Na current, blue; K current, green) and axial current (green).
(D) Net membrane current calculated as the sum of Na, K, and leak currents.
(E) Net axial current is the sum of inflowing and outflowing axial currents, describing where axial current is produced (negative values) or consumed (positive values).
(F) SDs of the currents in (C–E), color coding as above.
Cf. main text, Figure 3.
(1.7 MB PDF)

Figure S4 Variability of the Propagating AP Aligned at Half-Peak Membrane Potential (*t* = 0 ms, Data for 0.5 μm Diameter Axon, White/Black Curves Are Arithmetic Averages and Yellow Curves Are 3 × SD Envelopes, *N* = 673) (A) Membrane potential wave forms.
(B) Open Na (blue) and K (red) channels.
(C) Ionic currents flowing through the membrane (Na current, blue; K current, green) and axial current (green).
(D) Net membrane current calculated as the sum of Na, K, and leak currents.
(E) Net axial current is the sum of inflowing and outflowing axial currents, describing where axial current is produced (negative values) or consumed (positive values).
(F) SDs of the currents in (C–E), color coding as above.
Cf. main text, Figure 3.
(1.0 MB PDF)

Figure S5 Variability of the Propagating AP Aligned at Half-Peak Membrane Potential (*t* = 0 ms, Data for 1 μm Diameter Axon, White/Black Curves Are Arithmetic Averages and Yellow Curves are 3 × SD Envelopes, *N* = 673) (A) Membrane potential wave forms.
(B) Open Na (blue) and K (red) channels.
(C) Ionic currents flowing through the membrane (Na current, blue; K current, green) and axial current (green).
(D) Net membrane current calculated as the sum of Na, K, and leak currents.
(E) Net axial current is the sum of inflowing and outflowing axial currents, describing where axial current is produced (negative values) or consumed (positive values).
(F) SDs of the currents in (C–E), color coding as above.
Cf. main text, Figure 3.
(686 KB PDF)

Table S1 Parameter Variation in a 0.3 μm Diameter Squid Axon of 10 mm Length Effects of varying Na and K channel densities (r*_Na_*,r_K_), as well as varying axoplasmic resistance (*R_a_*) by ±50% from standard parameter values in Table S1. Table S1 lists spike time jitter and the occurrence of stochastic APs (SAP) and stochastic microsaltatory conduction effects, which occurred for all parameters. The term *noise* refers to the breakdown of communication on the axon, being so noisy such that no specific AP could be discerned.
(30 KB DOC)

Table S2 Parameter Variations for a 0.3 μm Diameter Cortical Pyramidal Cell Axon Collateral of 10 mm Length Effects of Varying Na and K Channel Densities (r*_Na_*,r*_K_*), Axoplasmic Resistance (*R_a_*), Membrane Leak (*g_Leak_*), and Resting Potential (*V*_0_) ±50% from Standard Parameter Values in Table S1
Change in spike time travel time jitter (measured in SD) and the occurrence of SAPs and stochastic microsaltatory conduction effects, which occurred for all parameters. The term *noise* refers to the breakdown of communication on the axon, being so noisy such that no specific AP could be discerned. The term *not excitable* refers to the AP being either not triggerable or not repolarizing.
(25 KB PDF)

Table S3 Full Parameter Set for Our Hybrid Axon Model Used in the First Protocol See main text for details.
(47 KB DOC)

Table S4 Full Parameter Set for the Squid Axon Model Used in the Second Protocol See main text for details.
(47 KB DOC)

Table S5 Parameter Set for a Cortical Pyramidal Cell Axon Collateral Used in the Second Protocol See main text for further details.
(46 KB DOC)

Table S6 Parameter Values for the Kinetic Functions of the [43] Na^+^ Channel Model (35 KB DOC)

Text S1 Supporting Information (154 KB PDF)

## Abbreviations

AP: action potential
SAP: stochastic AP
SD: standard deviation
