# Supplementary material for: Stochastic Simulations on the Reliability of Action Potential Propagation in Thin Axons
Source: PLoS Comput Biol. 2007 May 4;3(5):e79. doi: 10.1371/journal.pcbi.0030079 (PMC1864994; doi:10.1371/journal.pcbi.0030079)
Supplement: Supplementary file 1 [file pcbi.0030079.sd001.pdf]

Supporting Information

Insensitivity to ion channel kinetics and gating schemes

The key findings of our paper are based on the fact that channel gating is *per se* stochastic. Our extensive parameter variations demonstrate that the precise means by which channels generate noise and interact is of secondary importance, such as the independence of the channel gates, or the number of states. Thus the findings we present here are rather general. The novel effects that we report here depend on the following basic biophysical factors:

1. The general nature of the mechanisms by which Na channels regenerate (and thus) propagate the AP, namely; Na channels supporting the AP have to open earlier and faster then the opposing K channels. Na channel have to inactivate to allow for repetitive unidirectional signaling.

2. The fact that channels are discrete conductances. The input conductance of the membrane becomes smaller the thinner the axon, approaching that of single channels.

3. The fact that channels gate stochastically.

These simple facts suffice to explain the observed effects and their robustness (see also Tab. S1,S2).

We have tested our axon simulations with several Na channel models that differ with respect to activation/deactivation/inactivation kinetics, and gating schemes. We modeled squid axon using the Na channel model by Patlak (1991), where among other things, activation and inactivation are not independent. We observed the same stochastic effects as in the case of the standard squid m^3h model, supporting our finding that the details of the channel gating scheme are second order effects, with respect to AP propagation.

We also simulated the pyramidal cell axon using a model of hippocampal Na channels [44], which has more states than the cortical one. The more recently proposed models for cortical Na channels [45, 46] have not been included in our study because they are controversial (see discussion in Supplementary Material and also [47]) but, by increasing the rate at which the membrane potential changes just above threshold, they produce a greater decrease in spike time reliability then the models used here [46]. Thus, our data demonstrate an upper bound to the reliability of pyramidal cell axons.

Note, also that their steeper rising AP does not affect our findings, also because they consider AP initiation while we consider AP conduction/propagation; Due to the reaction-diffusion nature of membrane potential spread in cylindrical membranes any steep rise of membrane potential during AP initiation will be attenuated in the propagating AP - hence propagating APs will always have a broader rising foot then those generated *ab initio*.

We conclude that, as in our previous work, [19], where we showed that channel noise sets a lower limit to axon diameter that is remarkably insensitive to the specific details of the channel model, all novel stochastic effects on AP propagation we reported in this paper occurred independent of the channel gating model we used. Thus an extension to the latest gating models is unnecessary.

It is interesting that both the Naundorf model and Baranauskas model are based on the same cortical cell types and seem, therefore, to contradict each other in the details of their gating schemes. The Naundorf model uses cooperative gating, where the opening probability of Na channels increases, the greater the number of Na channels in their cluster that are already open. This positive feedback effect suggests that small fluctuations can increase the speed with which the members of a cluster of Na channels open. Thus the Naundorf Na channel is going to be more noisy that m^3h type channels. Baranauskas & Martina (2006) explicitly acknowledge that channel noise is higher in a single gate model in their discussion, and pose that as a problem for spike time reliability. Thus, these new models describe behavior that degrades reliability so that pyramidal cells will perform worse than we predict but, as we point out above, only slightly worse. Note also, that despite the fact that the Baranauskas model is suggested to have just a single gate, the model has two closed states and a single open state. It uses two gates, one with a slow and one with a very fast kinetic, i.e. it is a $m_{slow}\ m_{fast}\ h$ model.

In conclusion, the m^3h-type and the Patlak and Kuo & Bean type models we use will underestimate channel noise in comparison to both the model of Baranauskas & Martina and Naundorf et al. This means that our data suggest an upper bound to the reliability of pyramidal cell axons, but, as we argue above, the differences between our predictions and stochastic models based on these latest channel schemes must, from first principles, be small.

Stochastic squid axon model

Our standard squid axon model is based on the stochastic version model of Hodgkin and Huxley's original work [52]. We simply replaced the deterministic conductances by Markov channel models of Na and K conductances, as was previously done, [6, 7, 26, 9, 19].

However, to test the sensitivity with respect to detailed channel kinetics we also implemented a squid Na^+ channel model that takes into account the known problems of gating-particle based models. It is a Markov model with an asymmetric kinetic scheme [43] (Model 8 in the paper) and is based on patch clamp measurements of squid axon Na^+ channels. The model's kinetic scheme has 7 discrete states, of which states c_1 to c_4 are closed, o is the open state, and c_5 and c_6 are inactivated states (see Fig. S1).

The model ahas ten kinetic functions, named in this specific case $\alpha_m(v)$,$\beta_m(v)$,$\alpha_h(v)$,$\beta_h(v)$,$k_{ci}(v)$, $k_{ic}(v)$,$\delta(v)$,$\gamma(v)$,$\kappa(v)$ and $\lambda(v)$ and are defined by Eq.19-Eq.24. Note, that the kinetic functions $k_{ci}(v)$ and $k_{ic}(v)$ have the form of $\alpha_i(v)$ and $\beta_i(v)$. Here, v is the membrane potential in Volt, T denotes absolute temperature in Kelvin, k_B is the Boltzmann constant and h

Plank's constant. The model's fixed parameters are the following. Parameter z is the gating charge q in units of elementary charge of the proton. E_a is an activation energy in units of k_BT, d is a displacement and Δ a correction factor. The values of these parameters are listed in Tab. S6. Because by convention kinetic rate functions yield transition rates in units of 1/ms, the functions here are divided by 1000.

The kinetic functions are unbounded exponentials which can result in unrealistically large reaction rates and hence channel protein conformation changes. [43] suggested a limiting reaction rate of $R_{max} = 8ms^{-1}$ but conceded that this value was used because it worked. The fastest conformation changes in proteins, however, are known to occur several orders of magnitude faster [56]. This time scale mismatch prompted a review of the literature which produced the following result. A metabotropic ion channel in muscle was shown to switch open within at least 10 μs [57]. Measurement precision accounted for at least 2-3μs jitter and because voltage-gated ionotropic channels in neurons may switch faster than metabotropic channels in muscles we chose a limiting rate of $R_{max} = 333ms^{-1}$. Each kinetic function f was replaced by a rate-limited kinetic function f^* Eq.1 that allows for a smooth approach to the limiting rate.

$$f^*(V) = \frac{f(V)\ R_{max}}{f(V) + R_{max}} \tag{1}$$

The smoothing approach is advisable to prevent overshooting and ringing solution when solving the kinetic's differential equation with arbitrary steep non-linearities. The approach is physically justifiable as increasingly high gating speeds could be constrained by non-linear friction related losses.

Stochastic cortical pyramidal cell axon collateral model

Our model is the first pyramidal cell axon model that accounted for stochastic ion channels [19] and is based on published patch clamp measurements of axon properties. [55] patch-clamped layer 5 pyramidal cells from Sprague-Dawley rat neocortical slices and provided first electrophysiological data on axonal conductances past the axon hillock, near the region where axon collaterals branch of the central axon. Unfortunately several essential parameters to construct a full model of the axon were missing. Based on their figures, methods and drawing from other literature we constructed a full set parameters that should make a good model of axon collaterals (see [19] and reproduced here Tab. S5). The experiments were conducted at 23 C which is assumed to be the base temperature for all temperature-dependent parameters and kinetic functions. The figures in [55] suggest that the resting membrane potential of the cell was about -63 mV.

What Na^+ channel does this our channel model describe? Little combined electrophysiological and genetic data exists. We conducted a limited literature survey to match the *loci* where channels were patch-clamped with those where gene expression patterns of channels were studied, assuming that they describe the same channel. In the mammalian central nervous system Nav channel isoform are expressed to mediate the inward sodium current. Nav1.2 is expressed predominantly in unmyelinated axons of

the central nervous system, especially in mossy fibers of dentate granule cells, the hippocampus, and the molecular layer of the cerebellum [58, 59, 60]. Interestingly, it is also expressed in demyelinated axons [61]. In contrast Nav1.1 and Nav1.3 channel isoforms are localized mainly at the neuronal soma, especially that of dentate granule cells, hippocampal pyramidal cells, cerebellar Purkinje cells and spinal motor neurons [59]. Data on retinal ganglion cells having axons that are both unmyelinated and myelinated express Nav1.2 and Nav1.6 channel isoforms. The Nav1.2 isoforms, however, are found only in the unmyelinated axon segment and the Nav1.6 isoform is found only at Nodes of Ranvier of myelinated axon segment [62]. This suggests that our Na^+ channel model described by Eq.2-Eq.7 is that of the Nav1.2 channel isoform.

$$p_{Na}(V,t) = m(V,t)^3 h(V,t) \tag{2}$$

$$\frac{dm(V)}{dt} = \alpha_m(V)(1-m) - \beta_m(V)m \tag{3}$$

$$\alpha_m = 0.182 \frac{V+46.5}{1-\exp[\frac{-V-46.5}{6}]} \tag{4}$$

$$\beta_m = 0.124 \frac{-V-46.5}{1-\exp[\frac{V+46.5}{6}]} \tag{5}$$

$$\frac{dh(V)}{dt} = \alpha_h(V)(1-h) - \beta_h(V)h \tag{6}$$

$$\alpha_h = -0.015 \frac{V+69}{1-\exp[\frac{V+69}{6}]} \tag{7}$$

$$\beta_h = -0.015 \frac{-V-69}{1-\exp[\frac{-V-69}{6}]} \tag{8}$$

The question remains open whether the difference in voltage-dependence between somatic and axonal Na^+ channels described by [55] are related to differences between the Nav1.2 and Nav1.1, Nav1.3 isoforms. A combined biophysical and gene expression study is lacking. Experimentally this question could be easily resolved if outside-out patch-clamp measurements of channel kinetics were combined with a subsequent application of channel specific antibodies on the excised membrane.

We found that AP properties, such as duration and speed agreed well with published experimental data. As an alternative to the Na channel kinetics used here we also tested our data with a hippocampal cell Markov Na channel model which has 10 states [63] and found matching results.

Our model uses cortical pyramidal cell K^+ channels (putatively Kv1.1-Kv1.3) based on published patch-clamp data [64] and modeled as described in [19].

$$p_K(V,t) = n(V,t)^4 \tag{9}$$

$$\frac{dn(V)}{dt} = (n_\infty(V)^* - n)(1 - \exp[-t/\tau_n^*]) \tag{10}$$

$$\tau_n^* = 1.8ms \quad (11)$$
$$n_\infty^*(V) = \frac{\alpha(V)}{\alpha(V)+\beta(V)} \quad (12)$$
$$\alpha(V) = -0.0035\frac{V+30}{\exp\left[\frac{V+30}{-13}\right]-1} \quad (13)$$
$$\beta(V) = 0.0035\frac{V+30}{\exp\left[\frac{V+30}{13}\right]-1} \quad (14)$$

This original model was re-fitted to compute the kinetic functions required to model the Markov channel (see [19]):

$$p_K(V,t) = n(V,t)^4 \quad (15)$$

$$\frac{dn(V)}{dt} = \alpha_n(V)(1-n) - \beta_n(V)n \quad (16)$$
$$\alpha_n(V) = \frac{0.555556}{1+\exp[0.0769231(-30.0-v)]} \quad (17)$$
$$\beta_n(V) = \frac{0.555556}{1+\exp[0.0769231(30.0+v)]} \quad (18)$$

We compared both the resulting kinetic functions and the conductance kinetics to the published original model and found negligible differences $< 10^{-14}$ in the results. This allowed us then to construct the Markov channel model in a straightforward manner.This K^+ conductance completes the model of the cortical pyramidal cell axon collateral.
