# Supplementary material for: Stochastic Simulations on the Reliability of Action Potential Propagation in Thin Axons
Source: PLoS Comput Biol. 2007 May 4;3(5):e79. doi: 10.1371/journal.pcbi.0030079 (PMC1864994; doi:10.1371/journal.pcbi.0030079)
Supplement: Supplementary file 4 [file pcbi.0030079.sg005.pdf]

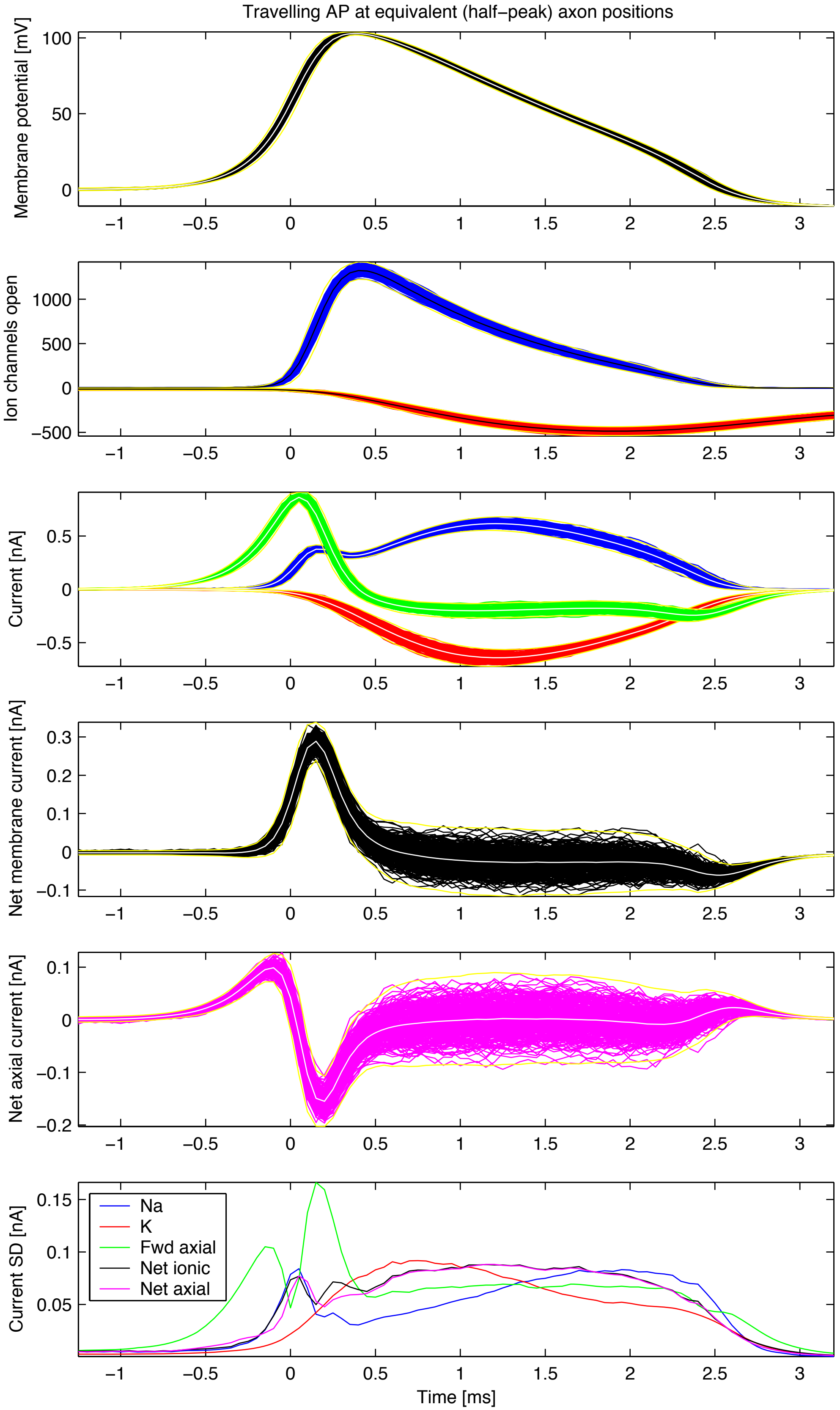
Travelling AP at equivalent (half-peak) axon positions
Membrane potential [mV]
Ion channels open
Current [nA]
Net membrane current [nA]
Net axial current [nA]
Current SD [nA]
Na
K
Fwd axial
Net ionic
Net axial
Time [ms]
