# Supplementary material for: Stochastic Simulations on the Reliability of Action Potential Propagation in Thin Axons
Source: PLoS Comput Biol. 2007 May 4;3(5):e79. doi: 10.1371/journal.pcbi.0030079 (PMC1864994; doi:10.1371/journal.pcbi.0030079)
Supplement: Supplementary file 5 [file pcbi.0030079.st002.pdf]

Jitter SD	SAP	Stoch. μ-saltation	
∞	*noise*	*noise*	
-10%	y	y	
-5%	y	y	
∞	*not excitable*	*not excitable*	
+21%	y	y	
∞	*noise*	*noise*	
+51%	y	y	
+12%	y	y	
+14%	y	y	
∞	*noise*	*noise*	
∞	*not excitable*	*not excitable*	
+498%	y	y	
+1016%	y	y	
-5%	y	y	

Table S2: Parameter variations for a 0.3 μm diameter cortical pyramidal cell axon collateral of 10mm length Effects of varying Na and K channel densities (ρ_{Na},ρ_K), axoplasmic resistance (R_a), membrane leak (g_{Leak}), and resting potential (V_0) ±50% from standard parameter values in Tab. SS2. The table lists the change in spike time travel time jitter (measured in SD) and the occurrence of stochastic APs and stochastic micro-saltatory conduction effects, which occurred for all parameters. The term *noise* refers to the break down of communication on the axon, being so noisy, such that no specific AP could be discerned. The term *not excitable* refers to the AP to be either not triggerable or not repolarizing.
