# Supplementary material for: Stochastic Simulations on the Reliability of Action Potential Propagation in Thin Axons
Source: PLoS Comput Biol. 2007 May 4;3(5):e79. doi: 10.1371/journal.pcbi.0030079 (PMC1864994; doi:10.1371/journal.pcbi.0030079)
Supplement: Supplementary file 6 [file pcbi.0030079.sg001.doc]

Figure S1: Markov state transition scheme of the Patlak Na\s\up6(+) channel model.
