# Supplementary material for: Stochastic Simulations on the Reliability of Action Potential Propagation in Thin Axons
Source: PLoS Comput Biol. 2007 May 4;3(5):e79. doi: 10.1371/journal.pcbi.0030079 (PMC1864994; doi:10.1371/journal.pcbi.0030079)
Supplement: Supplementary file 7 [file pcbi.0030079.st001.doc]

| Parameter | Jitter SD | SAP | Stoch. μ-saltation |
| --- | --- | --- | --- |
| $\rho_{Na}=90\mu m^{-2}$ | ∞ | *noise* | *noise* |
| $\begin{matrix} \rho_{Na}=90\mu m^{-2}, \\ \rho_{K}=27\mu m^{-2} \end{matrix}$ | +47% | y | y |
|  | +23% | y | y |
|  | +22% | y | y |
|  | +19% | y | y |
|  | -14% | y | y |
|  | +19% | y | y |
|  | -38% | y | y |

Table S1: Parameter variation in a 0.3 μm diameter squid axon of 10mm length. Effects of varying Na and K channel densities (,), as well as varying axoplasmic resistance () by ±50% from standard parameter values in Tab. SS1. The table lists spike time jitter and the occurrence of stochastic APs (SAP) and stochastic micro-saltatory conduction effects, which occurred for all parameters. The term *noise* refers to the break down of communication on the axon, being so noisy, such that no specific AP could be discerned.
