# Supplementary material for: Stochastic Simulations on the Reliability of Action Potential Propagation in Thin Axons
Source: PLoS Comput Biol. 2007 May 4;3(5):e79. doi: 10.1371/journal.pcbi.0030079 (PMC1864994; doi:10.1371/journal.pcbi.0030079)
Supplement: Supplementary file 8 [file pcbi.0030079.st003.doc]

| Parameter | Value | Unit | Source |
| --- | --- | --- | --- |
| Membrane |  | | |
|  | 1 |  | [Error: Reference source not found] |
|  | 20000 |  | [Error: Reference source not found] |
|  | 70a | Ω  *cm* | [Error: Reference source not found] |
|  | 0 | mV | [Error: Reference source not found] |
|  | 0.3 |  | see [Error: Reference source not found] |
|  | 10.613 | mV | [Error: Reference source not found] |
| Na current (i) |  |  | see [Error: Reference source not found, Error: Reference source not found] |
|  | 120 |  | [Error: Reference source not found] |
|  | 60 |  | see [Error: Reference source not found] |
|  | 20 | pS | [Error: Reference source not found] |
|  | 115 | mV | [Error: Reference source not found] |
|  | 6.3 | C | [Error: Reference source not found] |
|  | 3 |  | [Error: Reference source not found] |
|  | 3 |  | [Error: Reference source not found] |
| K current (i) |  |  | see [Error: Reference source not found, Error: Reference source not found] |
|  | 36 |  | [Error: Reference source not found] |
|  | 18 |  | see [Error: Reference source not found] |
|  | 20 | pS | [Error: Reference source not found] |
|  | -12 | mV | [Error: Reference source not found] |
|  | 6.3 | C | [Error: Reference source not found] |
|  | 3 |  | [Error: Reference source not found] |

Table S3: Full parameter set for our hybrid axon model used in the first protocol. See main text for details.
