# Supplementary material for: Stochastic Simulations on the Reliability of Action Potential Propagation in Thin Axons
Source: PLoS Comput Biol. 2007 May 4;3(5):e79. doi: 10.1371/journal.pcbi.0030079 (PMC1864994; doi:10.1371/journal.pcbi.0030079)
Supplement: Supplementary file 10 [file pcbi.0030079.st005.doc]

| Parameter | Value | Unit | Source |
| --- | --- | --- | --- |
| Membrane |  | | |
|  | 1 |  | [Error: Reference source not found] |
|  | 20000 |  | [Error: Reference source not found] |
|  | 70 | Ω *cm* | [Error: Reference source not found] |
|  | -65 | mV | [Error: Reference source not found] |
|  | 0.05 |  | see [Error: Reference source not found] |
|  | 15.5 | mV | see [Error: Reference source not found] |
| Na current (i) |  |  | Eq.8-Eq.14 |
|  | 101.8 |  | [Error: Reference source not found] |
|  | 68 |  | see [Error: Reference source not found] |
|  | 14.8 | pS | [Error: Reference source not found] |
|  | 115 | mV | [Error: Reference source not found] |
|  | 6.3 | C | [Error: Reference source not found] |
|  | 2.2 |  | [Error: Reference source not found] |
|  | 2.6 |  | [Error: Reference source not found] |
| K current (i) |  |  | Eq.21-Eq.24 |
|  | 15.8 |  | [Error: Reference source not found] |
|  | 8 |  | see [Error: Reference source not found] |
|  | 14 | pS | [Error: Reference source not found] |
|  | -12 | mV | [Error: Reference source not found] |
|  | 6.3 | C | [Error: Reference source not found] |
|  | 3 |  | [Error: Reference source not found] |

Table S5: Parameter set for a cortical pyramidal cell axon collateral used in the second protocol. See main text for further details.
