# Supplementary material for: Stochastic Simulations on the Reliability of Action Potential Propagation in Thin Axons
Source: PLoS Comput Biol. 2007 May 4;3(5):e79. doi: 10.1371/journal.pcbi.0030079 (PMC1864994; doi:10.1371/journal.pcbi.0030079)
Supplement: Supplementary file 11 [file pcbi.0030079.st006.doc]

| Kinetic function |  | *z* | *d* | Δ |
| --- | --- | --- | --- | --- |
|  | -19.0 | 2.45 | 0.6 |  |
|  | -22.35 | 2.45 | 0.6 |  |
|  | -26.5 | 0.3 | 0.5 |  |
|  | -22.4 | 0.3 | 0.5 |  |
|  | -21.0 | 2.45 | 0.55 |  |
|  | -28.05 | 2.45 | 0.55 |  |
| δ(*v*) | -19.0 | 2.45 | 0.6 | -1.8 |
| γ(*v*) | -23.9 | 2.45 | 0.6 | -1.8 |
| κ(*v*) | -21.32 | 2.45 | 0.6 | -1.8 |
| λ(*v*) | -27.08 | 2.45 | 0.6 | -1.8 |

Table S6: Parameter values for the kinetic functions of the [Error: Reference source not found] Na channel model.
